# Ultra-thin grating coupler for guided exciton-polaritons in WS_2_ multilayers

**DOI:** 10.1515/nanoph-2022-0791

**Published:** 2023-02-07

**Authors:** HyunHee Cho, Dong-Jin Shin, Junghyun Sung, Su-Hyun Gong

**Affiliations:** Department of Physics, Korea University, Seoul 02841, South Korea

**Keywords:** 1D photonic crystal, exciton-polariton, grating coupler, guided mode resonance, transition metal dichalcogenide, tungsten disulfide

## Abstract

An ultra-thin transition metal dichalcogenide (TMDC) layer can support guided exciton-polariton modes due to the strong coupling between excitons and photons. Herein, we report the guided mode resonance in an ultra-thin TMDC grating structure. Owing to the strong exciton resonances in TMDCs, a TMDC grating structure shows guided-mode resonance even at a thickness limit of ∼10 nm and is capable of realizing polaritonic dispersion in a monolithic grating structure. We investigated the polarization and thickness dependence of the optical dispersion relations of the tungsten disulfide (WS_2_) grating structure. In addition, we confirmed that the monolithic WS_2_ grating coupler can be used to couple the near-field guided exciton-polariton out into the far field. We believe that ultra-thin TMDC layers can facilitate sub-wavelength nanophotonic applications.

## 1 Introduction

The emergence of two-dimensional (2D) van der Waals materials, such as graphene, hexagonal boron nitride (hBN), and transition metal dichalcogenides (TMDCs), has opened up a new era in the study of electronics and photonics at the nanoscale [1–9]. Among others, TMDCs have been one of the promising material platforms for future applications in nanophotonics and fundamental research owing to their novel optical properties. With the chemical formula MX_2_ (M = Mo, W; X = S, Se), monolayer TMDCs are direct-bandgap semiconductors exhibiting atomic-scale thickness, strong excitonic resonance [10, 11], and spin-valley coupling [12]. Thus, various photonic applications have been demonstrated by integrating monolayer TMDCs into conventional photonic devices. For example, high-performance light-emitting devices [13], optical modulation [14], and photodetection [15] with monolayer TMDCs have been successfully achieved by exploiting their outstanding light–matter interactions.

Recently, it was proposed that monolayer and multilayer TMDCs have the potential for use as passive photonic elements. In the early stages, a TMDC multilayer with a thickness above 100 nm was utilized to demonstrate light guiding [16, 17] and Mie resonance [18]. More recently, light guiding has been reported even in a suspended monolayer WS_2_ in air [19]. In the case of a TMDC layer on a glass substrate, because of the imbalance of the surrounding refractive index, light guiding becomes possible for a thicker TMDC layer (>∼10 nm) [20]. Because the dispersion curve of the guided light in TMDC layers follows an anti-crossing behavior near the exciton resonance [21], it is referred to as a guided exciton-polariton mode. The unique properties of the guided exciton-polariton mode are revealed when excitons in the TMDC layers are excited using a pumping laser. The exciton in the exciton reservoir can relax to the guided exciton-polariton mode; thus, it is strongly influenced by the properties of pumped excitons, such as exciton density and polarization. For example, under circular-polarization excitation, the polariton exhibits valley polarization at room temperature [21]. It is also worth noting that the lasing action from the indirect bandgap transition in a 50-nm-thick WS_2_ disk structure was recently reported [22]. Therefore, previous demonstrations make TMDCs a promising material platform for nanophotonic devices and imply the possibility of monolithic integration between TMDC-based photonic devices.

However, the guided polariton existing in the bare TMDC layers is not allowed to couple out to the far field, except at the end of the layer, because of the large lateral momentum of the mode compared with that of propagating light in air. Thus, a near-field microscope or evanescent coupling method should be applied to access polariton modes [16, 17, 21]. In this study, we design and implement grating couplers in layered WS_2_ to access guided polariton modes using far-field light. The high-order diffraction in a one-dimensional (1D) WS_2_ grating allows the propagating light to easily couple to the far field. We first confirm with Fourier space reflection spectroscopy that the grating enables the far-field coupling of guided light through guided mode resonance. Further, angle-resolved spectroscopy results exhibit high sensitivity with respect to the thickness of WS_2_. Finally, the far-field coupling of the guided polariton mode generated by non-resonant pumping is demonstrated.

## 2 Results

### 2.1 Theoretical calculation for a WS_2_ grating structure

**Figure 1:**
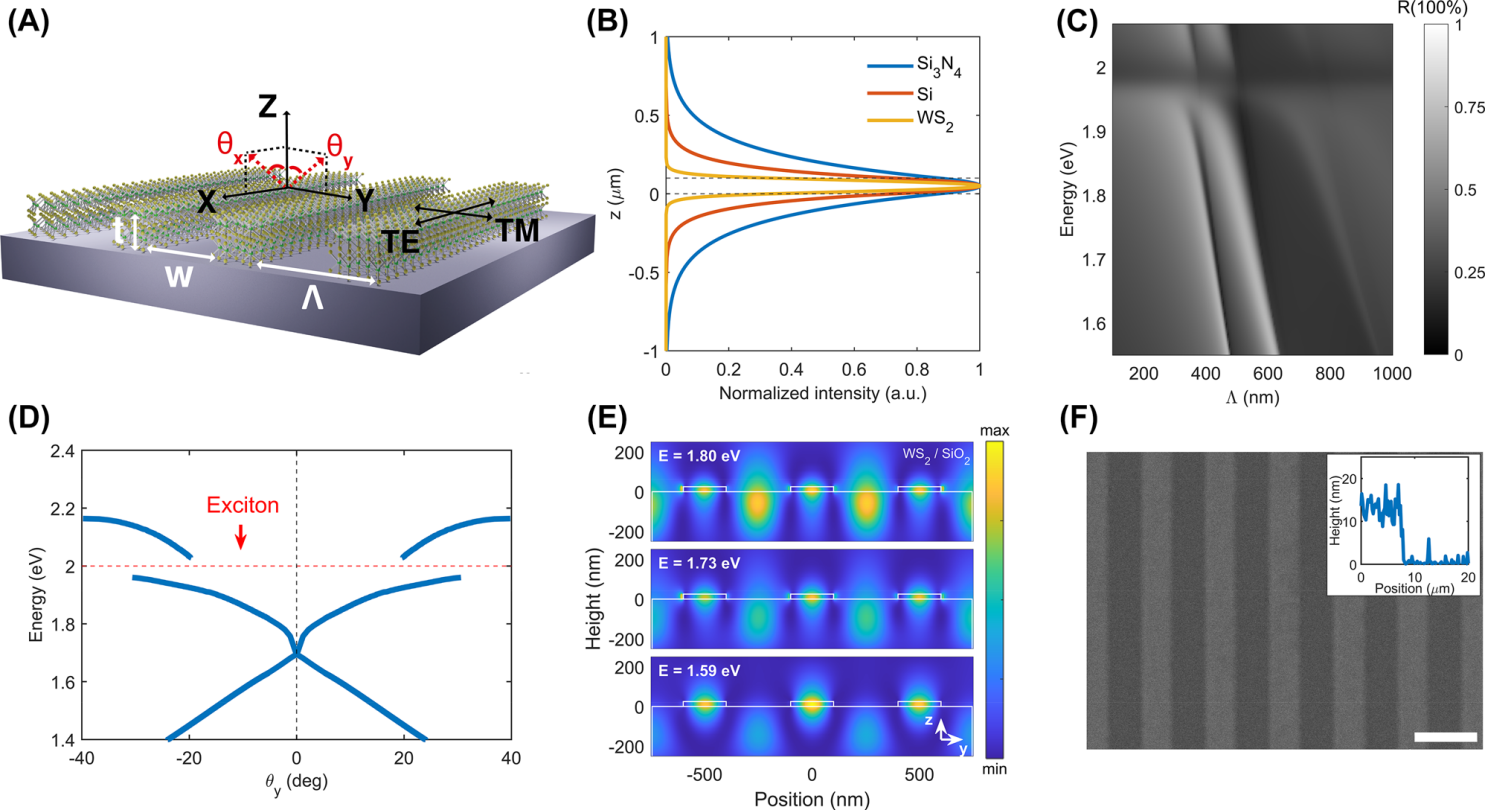
Characteristics of the one-dimensional WS_2_ grating. (A) Schematic structure of the grating. The design parameters are the thickness t of WS_2_, period Λ, and the width of WS_2_ in one period. (B) Normalized intensity of slab waveguides with different materials (thickness of 100 nm). (C) An example of reflectance spectra with variation of period Λ at *θ* = 12° (*t* = 20 nm). (D) Calculated optical dispersion relations for TE-polarized light (*t* = 20 nm, Λ = 500 nm, *F* = 0.4). The anti-crossing behavior near exciton resonance (1.97 eV) indicates strong light–matter interaction, and the spatial periodicity in the *y*-direction contributes to the dispersion folding. (E) Cross-section of simulated TE-polarized electric field profiles of the grating in y–z plane corresponding to different energies. (F) An SEM image of the grating on SiO_2_/Si substrate. Inset shows height profile of the WS_2_ layer measured by AFM. Scale bar = 500 nm.

Figure 1A shows a schematic of a 1D photonic crystal structure (i.e., grating structure) made of layered WS_2_. Among TMDC materials, WS_2_ is chosen because it shows a large oscillator strength of A exciton and high exciton resonance energy (∼1.98 eV). This makes a WS_2_ layer promising polariton waveguide in a wide spectral range at visible wavelengths. The thickness of the WS_2_ grating structure was designed to support guided exciton-polariton modes in the WS_2_ layer. Owing to the periodic modulation of the grating structure, far-field light can couple to the guided exciton-polariton mode. WS_2_ material has strong excitonic resonances and a large background permittivity, not only for monolayers but also for multilayers. Therefore, compared with conventional dielectric and semiconductor waveguides such as silicon (Si) and silicon nitride (Si_3_N_4_), TMDC waveguides can provide very strong mode confinement, as shown in Figure 1B. The thickness limit of the WS_2_ layer for the guided exciton-polariton modes is ∼7 nm when the WS_2_ layer is situated on a SiO_2_ substrate, which is comparable to the thickness of the plasmonic waveguides.

To design a grating structure with layered WS_2_, we characterized the following parameters: thickness *t*, period Λ, unit width *w*, and filling factor *F* = *w*/Λ. The guided mode resonance energy and angle are determined by the design parameters *t*, Λ, and *F* according to the phase-matching condition. Figure 1C shows the reflectance spectrum for a sample at *t* = 20 nm with an incident angle of *θ*_inc_ = 12°. We calculated the angle-resolved reflection data of grating structures by sweeping the period Λ from 100 nm to 1000 nm. We can see that guided mode resonances exist when Λ is approximately 400–650 nm.

Figure 1D shows an example of the optical dispersion relation of the WS_2_ sample where *t* = 12 nm, Λ = 500 nm, and *F* = 0.4. A typical feature of guided mode resonance, that is, the folded dispersion curve of the guided mode, was observed. One can also notice anti-crossing behavior near the exciton energy, indicating a strong coupling regime between excitons and photons. The vacuum Rabi splitting energy is approximately 100 meV. The large Rabi splitting energy is due to the monolithic structure of the WS_2_ grating, which leads to a large wavefunction overlap between the guided mode resonance and the exciton [23]. Figure 1E shows the TE-polarized electric field distribution in the y–z cross-section. The electric-field profiles confirm that guided mode resonance is formed in a very thin WS_2_ grating structure.

Based on the calculation results, we fabricated a grating pattern on multilayer WS_2_ using an e-beam lithography technique, followed by reactive ion etching. Patterned WS_2_ structures were placed on a SiO_2_/Si substrate with a SiO_2_ thickness of 280 nm. Figure 1F shows a scanning electron microscope (SEM) image of the fabricated 1D grating structure. The inset in Figure 1F shows the atomic force microscope (AFM) scanning profile of the WS_2_ grating. The minimum thickness of the fabricated WS_2_ grating structure was 10 nm, which is approximately 50 times smaller than the wavelength of light.

### 2.2 Reflection measurement for WS_2_ grating structure

First, we measured the optical dispersion relation of 1D photonic crystals made of WS_2_ using an angle-resolved microscopy setup to analyze the guided mode resonance. A supercontinuum laser was used as the white light source for reflectance measurement. A homemade Fourier plane optical microscopy and spectroscopy setup was used to measure the angle-resolved spectrum [23]. The polarization of incident light along the grating bars is defined as transverse electric (TE) and across the grating bars as transverse magnetic (TM), according to the coordinates described in Figure 1A.

**Figure 2:**
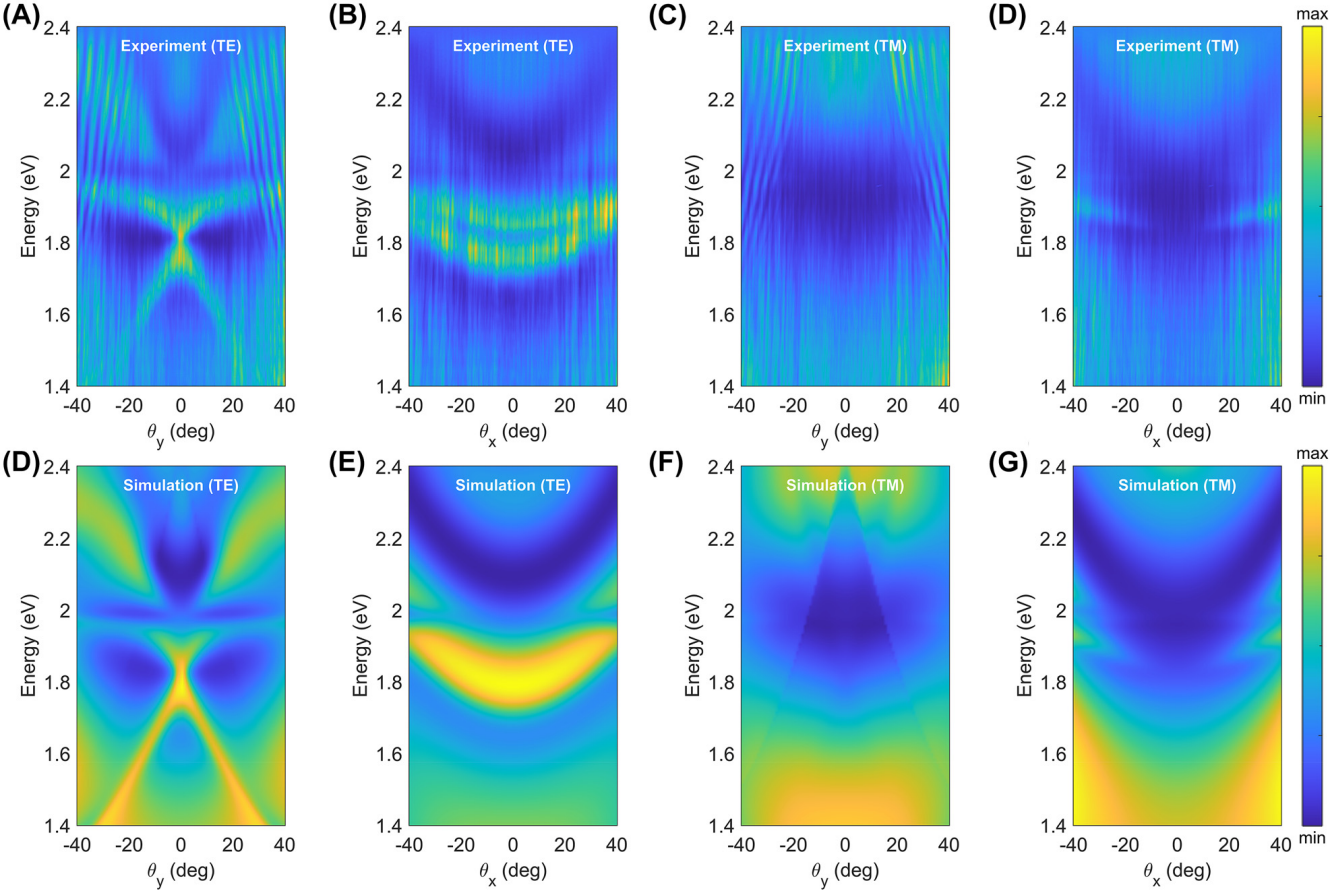
Angle-resolved reflectance spectra of the one-dimensional WS_2_ grating (*t* = 15 nm, Λ = 500 nm, *F* = 0.4). The top/bottom row shows the measured (A–D)/simulated results (E–H), respectively. (A, B) and (E, F) The angle-resolved reflectance spectra of the grating for TE-polarized incident light. The spectra are sensitive to direction of measurement due to the spatial anisotropy of the grating. (C, D) and (G, H) The angle resolved reflectance spectra of the grating for TM-polarized incident light.

For TE-polarized incident light (Figure 2A, B and E, F), the guided mode resonant feature was clearly seen as an intensity peak in the reflectance spectra, which matches well with the simulation results. The guided mode resonance observed in the reflectance spectra was the result of interference between the reflected light from two distinct pathways: one is directly reflected from the WS_2_ layer, and the other is reflected after the guided mode resonance takes place [8, 9]. Owing to the spatial anisotropy of the 1D photonic crystal structure, the optical dispersion relations exhibit propagation direction dependency (i.e., *k*_*x*_ vs. *k*_*y*_ or *q*_*x*_ vs. *q*_*y*_). In *y*-direction, where the translational symmetry is broken, there is a steep folded dispersion of the guided mode in the WS_2_ waveguide. In contrast, a parabolic dispersion is visible in *x*-direction, where translational symmetry holds. Importantly, for both *x* and *y* directions, we can observe anti-crossing behavior near the exciton energy of WS_2_. However, for TM-polarized incident light (Figure 2C, D and G, H), there is no guided mode resonance because the layer is too thin to support TM-guided modes. The parabolic shape appeared as the intensity deepened in the reflection spectra owing to the Fabry–Perot modes in a finite thickness of the SiO_2_ layer (280 nm).

**Figure 3:**
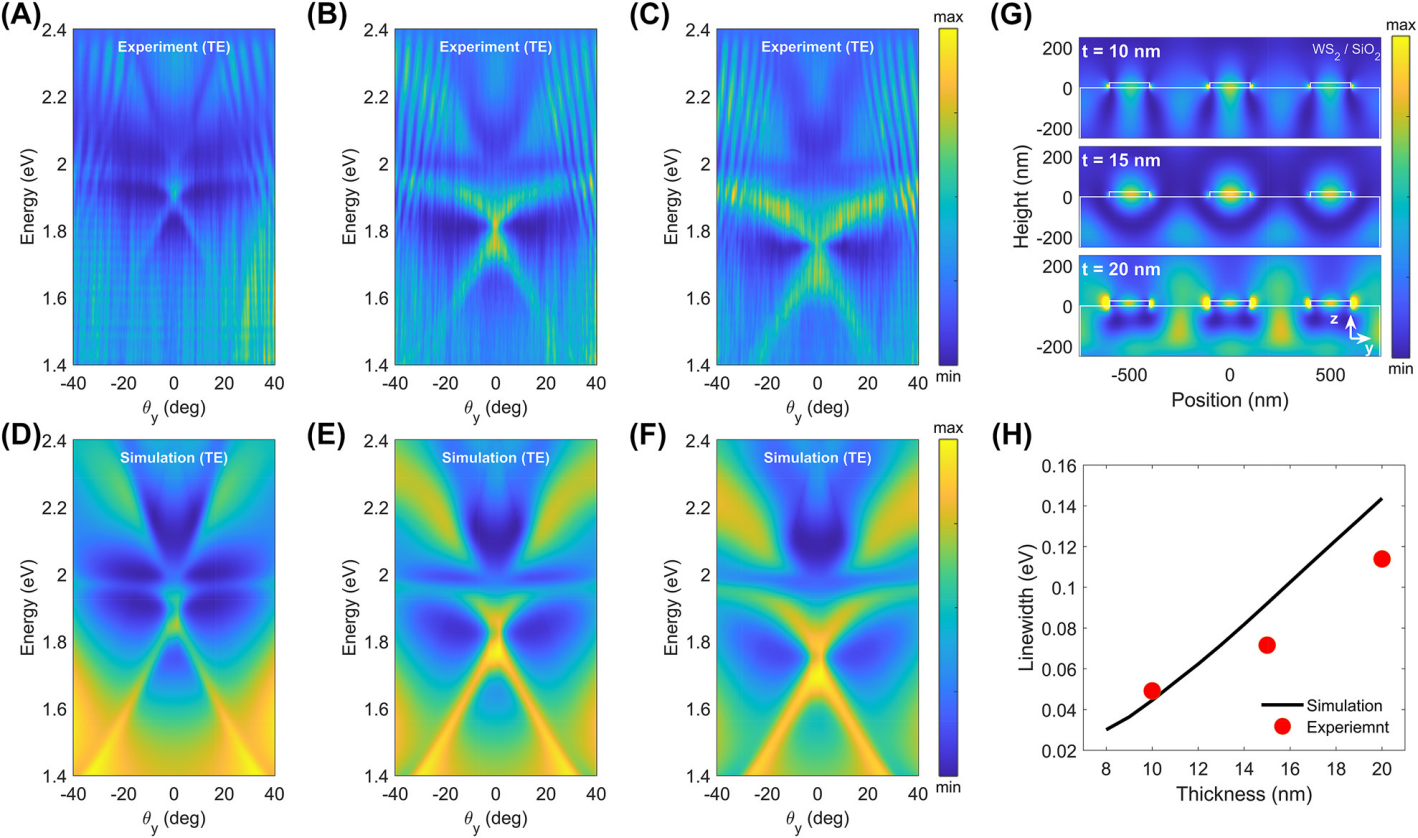
Angle-resolved reflectance spectra of the one-dimensional WS_2_ grating for different thicknesses (Λ = 500 nm, *F* = 0.4). The top/bottom row of (A–F) shows the measured (A–C)/simulated (D–F) results, respectively. (A–C) Angle-resolved reflectance spectra of the grating for *t* = 10 nm, 15 nm, and 20 nm, respectively. The redshift of resonance peaks and the broadened linewidth of peaks in the spectra are attributed to the increased effective index of the grating modes with increasing thickness. (G) Cross-section of simulated TE-polarized electric field profiles of the grating in y–z plane corresponding to different thicknesses with an energy of 1.61 eV. (H) Simulated and measured linewidth of resonance peaks for different WS_2_ grating thicknesses at *θ* = 10°.

We investigated the thickness dependence of the optical dispersion relations. WS_2_ grating structures were fabricated with thicknesses of 10, 15, and 20 nm, with the period Λ and filling factor *F* fixed. Figure 3 shows the measured optical dispersion relations for various thicknesses of the WS_2_ grating structures. As the sample thickness increased, the guided-mode resonance peaks shifted to lower energies and the widths of the peaks broadened. This was attributed to the stronger confinement of the guided modes in thicker layers. Figure 3D shows the calculated electric field distributions of the guided mode resonance. For thicker samples, the electric fields were highly confined in the WS_2_ material, which appeared as a higher effective refractive index of the guided modes. The increased real part of the effective mode index is shown as a redshift of the resonance peak, and the increased imaginary part is shown as broadening of the dispersion (Figure 3A–C). Figure 3E presents the calculated linewidth of the guided mode resonance as a function of thickness at a fixed angle of 10°. The experimental results agree well with the calculation results, which showed a broadening of the linewidth with increasing thickness. We also emphasize that an anti-crossing behavior near the exciton energy (∼1.97 eV) was observed for all thicknesses. The thickness-dependent optical dispersion results suggest broad controllability of the guided mode resonance in the WS_2_ grating.

### 2.3 Photoluminescence of WS_2_ grating structures under non-resonant pumping

**Figure 4:**
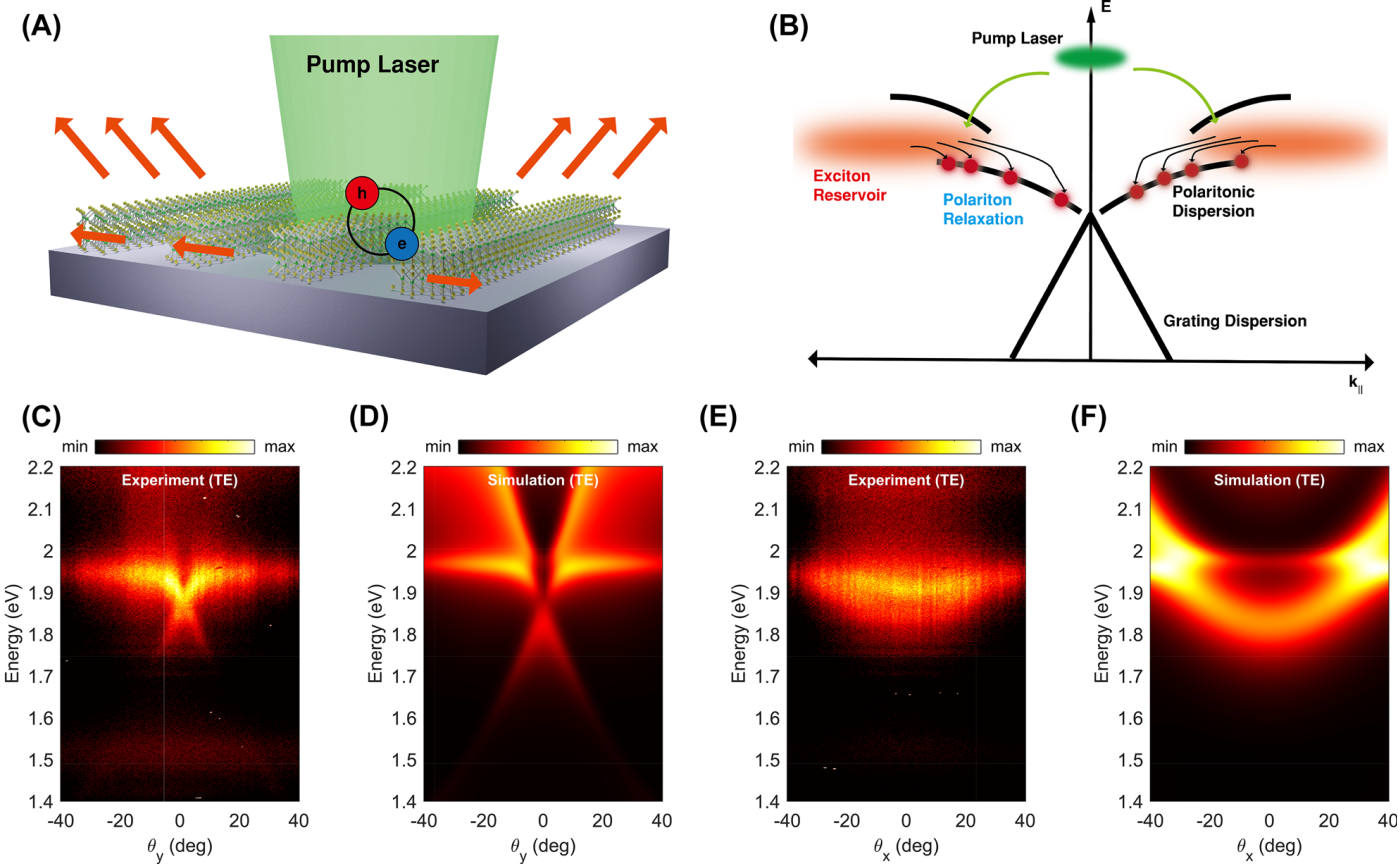
Angle-resolved PL spectra with non-resonant pumping. (A) Schematic illustration of the angle-resolved PL measurement with pumping laser (514 nm). (B) Schematic process of the relaxation of exciton-polaritons. Excitons in the exciton reservoir pumped by non-resonant laser pumping subsequently relax to polariton states via scattering processes. (C and D) Measured and simulated angle-resolved PL spectra for *θ*_*y*_ with TE-polarized pumping laser. (E and F) Measured and simulated angle-resolved PL spectra for *θ*_*x*_ with the same pumping laser.

We further analyzed the optical dispersion curve of the grating structure under non-resonant pumping conditions. To excite excitons at the direct bandgap of the WS_2_ layer, we used a 514 nm continuous-wave (CW) diode laser. The laser was focused on the middle of the grating structure with a pumping spot size of approximately 1 μm, as shown in Figure 4A. The photoluminescence (PL) process in a WS_2_ grating structure is different from that of an emitter in an optical grating system. When an emitter emits light in a conventional grating structure, the radiation rate is affected by the presence of the grating structure, which is known as the Purcell effect. Consequently, the radiation pattern of the emitter was modified according to the modified optical density of state with the grating structure.

In the case of our WS_2_ grating structure, excitons are directly relaxed from the excited states to the polaritonic guided mode resonance states, rather than having a modified radiation process with the Purcell effect. Figure 4B shows a schematic of the PL process of the exciton-polariton modes under non-resonant excitation pumping. Most carriers excited by the pumping laser accumulate at the exciton reservoir. The excitons in the exciton reservoir can then relax into the polaritonic dispersion branch. The polaritons at the guided mode resonance can couple to the far field, which can be observed using a far-field microscope.

Figure 4C shows the angle-resolved PL of the WS_2_ grating with a thickness of 10 nm. Bright emission at the guided mode resonance was observed near the WS_2_ exciton energy (1.97 eV). Owing to the thermal relaxation process, the emission of the lower polariton branch is brighter than that of the upper polariton branch. In addition, the intensity of the lower polariton branch decreases when the polariton energy is far from the exciton energy. This is attributed to both the large energy difference from the exciton reservoir and the limited density of states of the polariton modes that reduce the relaxation probability for the polariton occupation [21]. Moreover, unlike in the weak coupling regime, the background emission not coupled to the grating modes is not visible. There also exists slight emission in the 1.4–1.6 eV range, which originates from indirect bandgap transition [21]. For the PL dispersion of the TM-polarized mode, we could not see the momentum-dependent dispersion, which corresponds to the result in Figure 2C, D. The polaritonic dispersion measured with PL agrees well with the reflectance measurement. Note that the double parabolic modes observed in the reflection measurement (Figure 2B) were not observed in the PL measurement (Figure 4E). This implies that such double parabolic branches in the reflection data may results from interference between the guided mode resonance and background signals. We also verified that the polaritonic dispersion can be predicted by and agrees well with the simulation by calculating the absorption of the resonance mode of the WS_2_ grating (Figure 4D–F).

### 2.4 Far-field coupling of guided exciton-polariton at a WS_2_ grating

**Figure 5:**
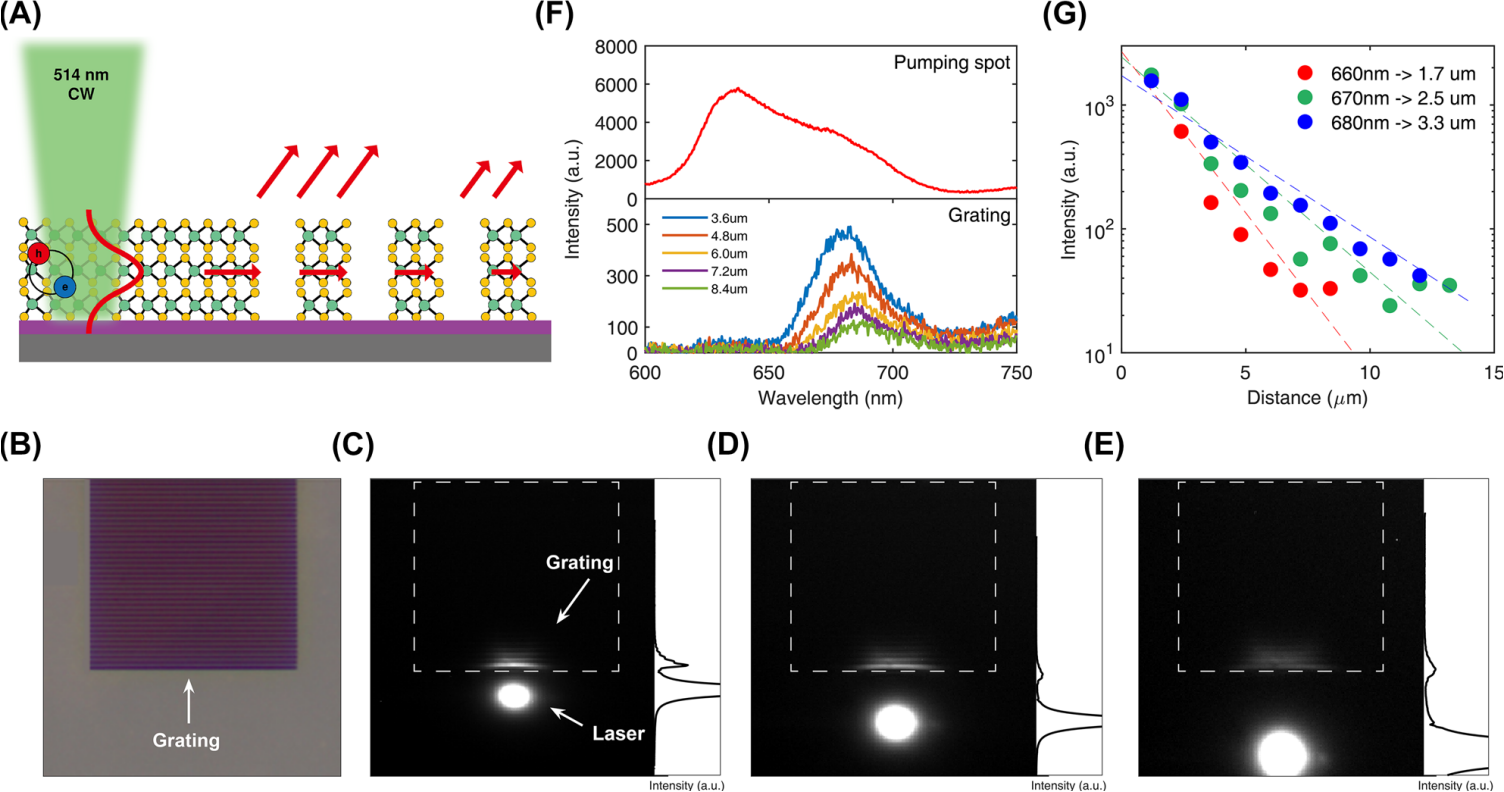
Far-field coupling of guided exciton-polaritons at the WS_2_ grating. (A) Schematic side view of the WS_2_ grating and pumping laser. (B) An optical microscope image of the grating (*t* = 15 nm, Λ = 500 nm, *F* = 0.4). The scale bar is 5 μm. (C–E) Show images of the laser spot and coupled guided mode on CCD camera with different distances between the laser spot and edge of the grating. The distances are 4.8 μm, 7.2 μm, and 9.6 μm, respectively. The right panels show the cross-section of the intensity profile in the *y*-direction (along with laser centered on the grating). (F) Spectrum of the pumping laser and the guided mode. Top: spectrum at the center of the laser spot. Bottom: spectrum of the guided mode coupling at the edge of the grating for different distances. (G) Intensity of the coupled exciton-polariton (wavelength of 660, 670, and 680 nm) with different distances between the laser center and the edge of the grating. The dashed lines indicate fitting lines for each wavelength.

Finally, we demonstrated that the patterned WS_2_ layer can act as an efficient grating coupler for guided exciton-polaritons. A CW laser with a wavelength of 514 nm was used for non-resonant excitation of the guided exciton-polariton modes in the non-patterned region of WS_2_ (Figure 5A). The excitation spot was placed a few micrometers away from the 1D grating coupler. The distance between the grating coupler and pumping spot was gradually increased from 2.4 μm to 12.8 μm. All measurements were conducted under the same excitation power.

Figure 5C–E shows the charge-coupled device (CCD) images with pumping spots at 2.4 μm, 6 μm, and 12.8 μm apart from the grating coupler, respectively. Strong emission is visible at the pumping spot, indicating the direct radiation from the exciton. In addition, scattered light was observed at the position of the grating coupler. This scattered light suggests that the guided polariton modes were coupled out from near-field to far-field with assistance from the grating coupler. This far-field scattering of the guided polariton mode was observed only for TE polarization.

To further distinguish between the scattered light from the grating and the direct radiation of the exciton at the pumping spot, we compared the spectrum measured at different positions. The upper graph in Figure 5F shows the spectrum at the pumping spot, with an exciton peak at a wavelength of ∼630 nm. The lower graph shows the distance-dependent spectrum measured at the grating position. Because the polaritons occupied the lower polariton branch, the polariton spectrum was located at a longer wavelength than the exciton spectrum. Moreover, as the distance increased, the spectral intensity decreased with a continuous red-shift of the peak. This is attributed to the wavelength-dependent loss of polariton modes. As noticeable from the linewidth of the dispersion relations of the polariton modes, the polaritonic modes with higher energy show higher optical loss owing to exciton absorption. The red-shift of the spectrum as a function of propagation distance shows direct evidence of the far-field coupling of the guided polariton mode instead of direct excitation of the laser in the grating coupler.

Figure 5G shows the intensity of the spectrum as a function of propagation distance at various wavelengths. We fitted these experimental data with a single exponential function to estimate the propagation distance of the polaritons. The estimated propagation distances for the guided polaritons were 1.7, 2.5, and 3.3 μm at 660, 670, and 680 nm wavelengths, respectively. As demonstrated here, a grating coupler is useful for examining the fundamental properties of guided polariton modes in WS_2_ layers. If we collect the scattered guided polariton modes at the end of a bare layer, the collection efficiency is very low because the scattered light loses the momentum information of the guided polariton modes. In contrast, the coupled light from the grating coupler shows the folded polariton dispersion curves. Therefore, a grating coupler is a useful element to investigate the non-radiative polariton modes in the far-field [24, 25].

## 3 Conclusions

We demonstrated the guided mode resonance of an exciton-polariton in an ultra-thin WS_2_ grating structure. The strong exciton resonance of the WS_2_ layers results in polaritonic dispersions in the 1D grating structures. Because of the sub-wavelength confinement of the guided exciton-polariton mode in the WS_2_ layer, the guided mode resonance can be formed even in a 10-nm-thick grating structure. The optical dispersion relations of the grating structure were directly investigated using a Fourier-plane microscope. We also demonstrated far-field coupling of the guided exciton-polariton in a grating coupler. The grating coupler plays an important role in accessing the guided polariton modes in WS_2_ layers using a conventional spectroscopy setup. Note that other TMDC-grating structures exhibit similar behaviors because they also have strong exciton resonances. Confining and manipulating light based on guided mode resonance in ultra-thin TMDC layers can be used in applications such as optical filters, nonlinear optics, and sensors. We believe that our work demonstrates the great potential of a TMDC layer for future sub-wavelength nanophotonic applications.

## 4 Materials and methods

### 4.1 Theoretical modeling

The finite-difference frequency domain method developed in MATLAB was used to calculate the electric field profile of the slab waveguides of Si_3_N_4_, Si, and WS_2_. Electric field distributions around grating structures were calculated by Finite-difference time-domain method (FDTD, Lumerical). Simulations for the reflection and PL spectra of the grating was carried out using Rigorous Coupled-Wave Analysis (RCWA) developed in MATLAB. The refractive indices of SiO_2_ and Si are set as 1.46 and 3.85, respectively. The in-plane permittivity of WS_2_ was obtained from the Lorentz oscillator model as follows:

(1) $$\varepsilon \left(E\right)=\varepsilon_{\infty }+\underset{j}{\sum }\frac{f_{j}}{{E_{j}}^{2}-E^{2}-i\gamma_{j}E}$$

Here, we used four oscillators: exciton resonance energy *E*_*j*_ = 1.97, 2.4, 2.69, 2.95 eV, oscillator strength *f*_*j*_ = 0.53, 1.38, 3.18, 30 eV^2^, linewidth of the oscillator *γ*_*j*_ = 0.04, 0.18, 0.16, 0.52 eV and background permittivity *ε*∞ = 12 [21].

### 4.2 Sample preparation

Multilayer WS_2_ flakes were mechanically exfoliated from purchased bulk WS_2_ crystals (2D Materials). The grating patterns was fabricated by standard electron-beam lithography (EBL) process followed by reactive ion etching (RIE).

### 4.3 Optical spectroscopy setup

Optical measurements were performed using a home-built microscope with 50× objective of NA = 0.75. To characterize the optical dispersion relation of patterned multilayer WS_2_, angle-resolved spectroscopy was performed using a Fourier imaging setup. For reflection measurements, a white laser (supercontinuum laser, NKT Photonics) with a beam diameter of 15 μm was focused on the patterned multilayer WS_2_. For PL measurements, a continuous-wave diode laser with a wavelength of 514 nm with a beam diameter of 2 μm was focused to excite carriers in the sample. The polarization of the excitation laser and the collected light was controlled by half-waveplates. To avoid errors arising from polarization-dependent collection efficiency of the optical setup, a fixed linear polarizer was placed in front of the detection system.
